# Supplementary material for: An abscisic acid-responsive protein interaction network for sucrose non-fermenting related kinase1 in abiotic stress response
Source: Commun Biol. 2020 Mar 26;3:145. doi: 10.1038/s42003-020-0866-8 (PMC7099082; doi:10.1038/s42003-020-0866-8)
Supplement: Supplementary file 1 — Description of Additional Supplementary Files [file 42003_2020_866_MOESM1_ESM.pdf]

Supplementary Data

Supplementary Data 1. List of all 565 SnRK1-SnIP candidate interactions identified in the primary Y2H screen.

Supplementary Data 2. List of 281 curated SnRK1-SnIP high confidence interactions (HCI).

Supplementary Data 3. SnRK1 complex interactors identified in large-scale interaction studies.

Supplementary Data 4. List of 48 core SnIPs.

Supplementary Data 5. Cytoscape BINGO plugin p-values and biological process categories for the 125 curated SnRK1 interactors.

Supplementary Data 6. Pearson Correlation Coefficients (PCC) for the 48 core SnIPs network under abiotic stress.

Supplementary Data 7. Raw data for seedling establishment/growth values for Figure 6a, Figure 6b and Figure 8.

Supplementary Data 8. SnRK1/SnRK3 interaction network node and clustering information from Cytoscape
