# Supplementary material for: An abscisic acid-responsive protein interaction network for sucrose non-fermenting related kinase1 in abiotic stress response
Source: Commun Biol. 2020 Mar 26;3:145. doi: 10.1038/s42003-020-0866-8 (PMC7099082; doi:10.1038/s42003-020-0866-8)
Supplement: Supplementary file 2 — Supplementary Information [file 42003_2020_866_MOESM2_ESM.pdf]

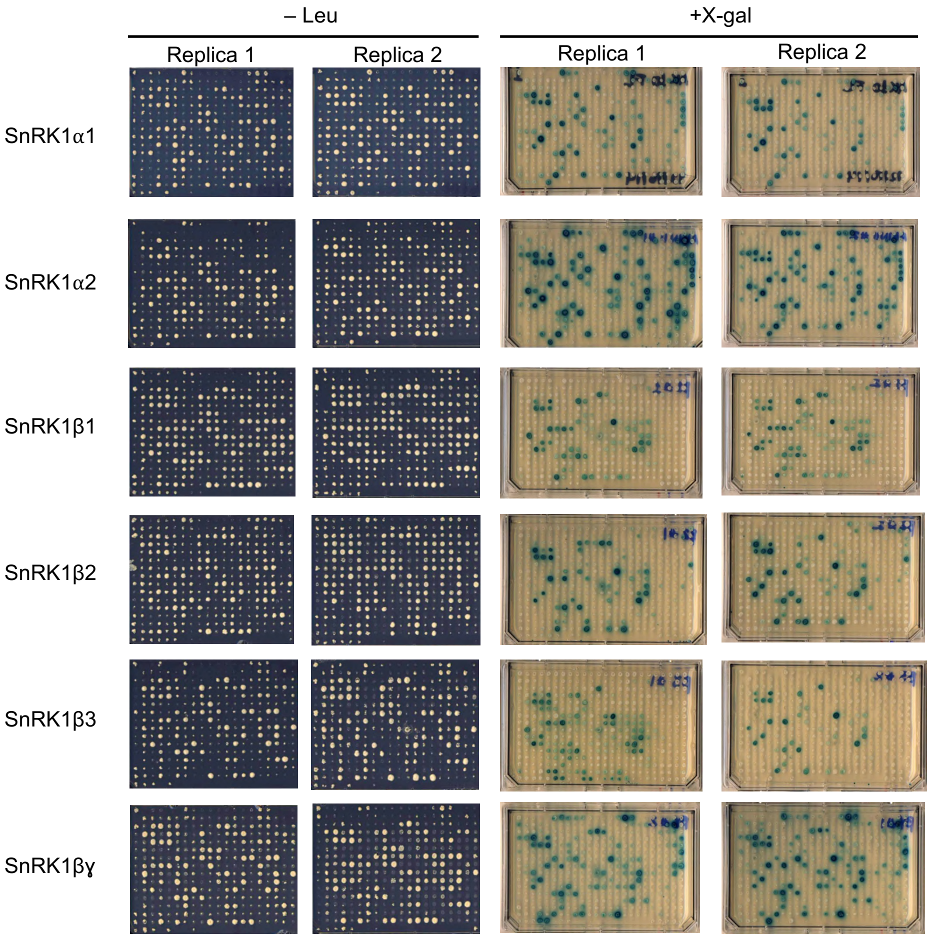

Supplementary Fig. 1. High-throughput Y2H interaction assay

Images of the plates showing colony growth on – Leu and blue colonies on + X-gal selection plates four days after mating. Ten colonies harbouring the AD empty vectors were included in each plate as the negative controls.

a

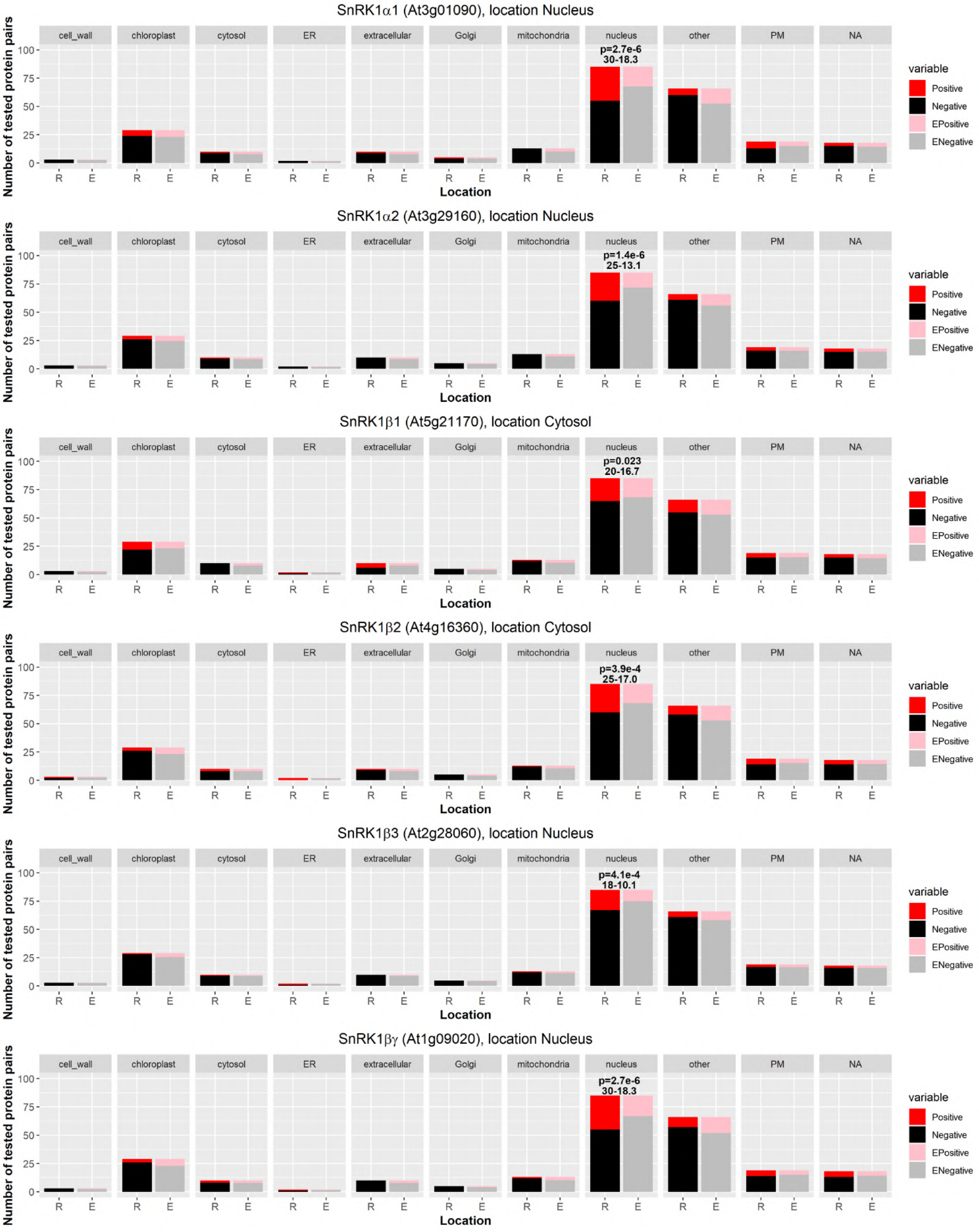
SnRK1α1 (At3g01090), location Nucleus
p=2.7e-6
30-18.3
SnRK1α2 (At3g29160), location Nucleus
p=1.4e-6
25-13.1
SnRK1β1 (At5g21170), location Cytosol
p=0.023
20-16.7
SnRK1β2 (At4g16360), location Cytosol
p=3.9e-4
25-17.0
SnRK1β3 (At2g28060), location Nucleus
p=4.1e-4
18-10.1
SnRK1βγ (At1g09020), location Nucleus
p=2.7e-6
30-18.3
cell_wall
chloroplast
cytosol
ER
extracellular
Golgi
mitochondria
nucleus
other
PM
NA
Number of tested protein pairs
Location
R
E
0
25
50
75
100
variable
Positive
Negative
EPositive
ENegative

b

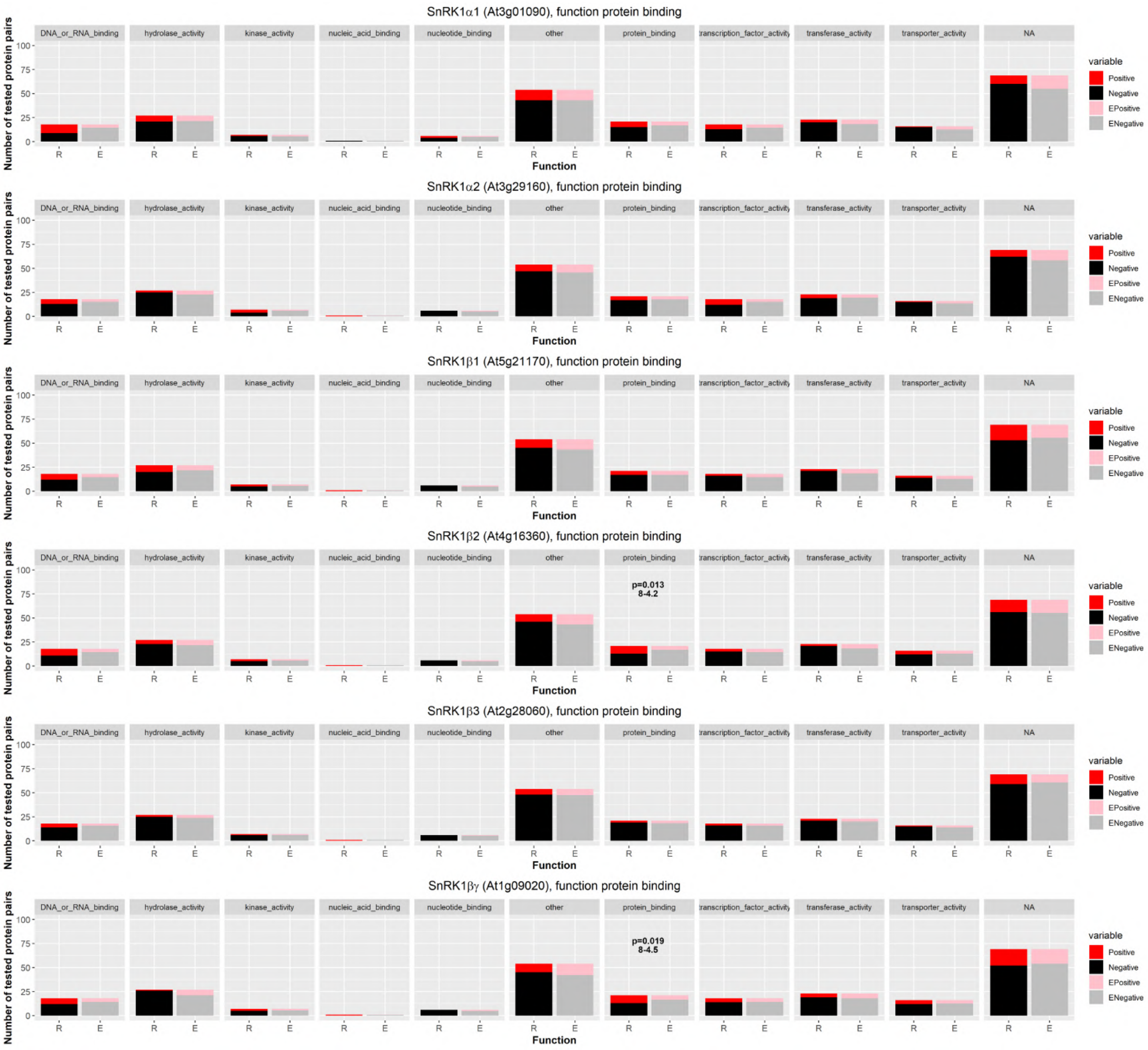

Supplementary Fig. 2. Co-molecular function and co-cellular localization enrichment analysis

a) The cellular localization and **b)** the molecular function enrichment status of each subunit is represented on each row. For all possible subcellular localizations from the GO Slim file, there are two bars, the one on left indicating the real numbers ("R", with red indicating Y2H positive interactions and black indicating Y2H negative interactions) and the one on right indicating the expected numbers ("E", with pink indicating Y2H positive interactions and grey indicating Y2H negative ones). If the height of the red bar is greater than the height of the pink bar, there is enrichment. If this enrichment is statistically significant, it is indicated along with its p-value.

a

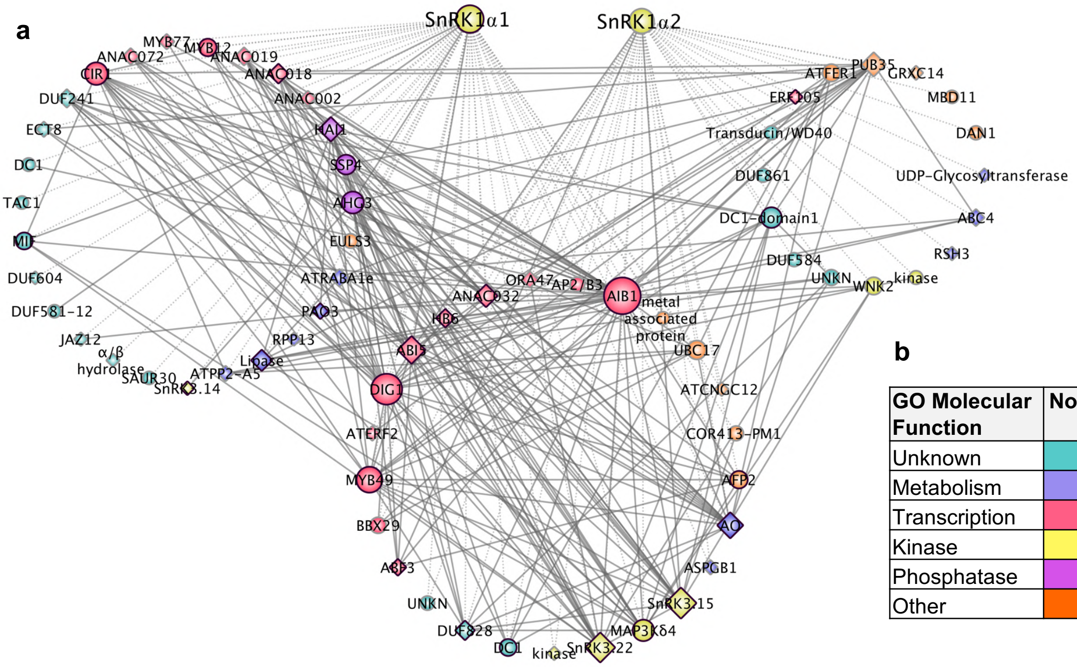

b

GO Molecular Function	Node	α1 (50)	α2 (40)	α1 & α2 (68)
Unknown		25%	20%	28%
Metabolism		13%	12%	15%
Transcription		35%	30%	28%
Kinase		10%	13%	9%
Phosphatase		6%	0%	4%
Other		12%	25%	16%

c

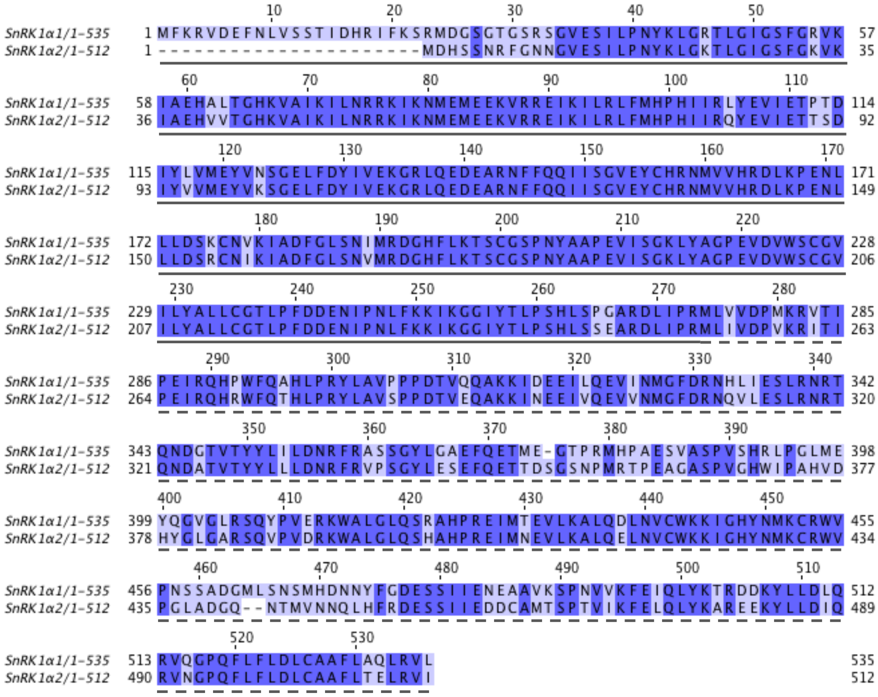

d

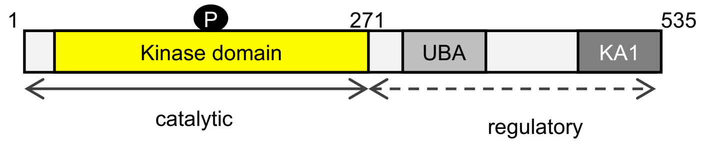

Supplementary Fig. 3. Overview of SnRK1α interactors

a 68 total SnRK1α interactors identified by Y2H are shown, 50 of which partner with SnRK1α1 (left), 40 with SnRK1α2 (right) and 25 are shared (center). Interactors are shown as nodes and coloured based on GO molecular function. Edges indicate protein-protein interactions; dotted lines are interactions from this study; solid lines indicate published interactions obtained from the Arabidopsis Interaction Viewer. **b** Percentage distribution by GO molecular function for each set of α-interactors. **c** ClustalOmega alignment of SnRK1α1 and α2 subunits showing divergence at the C-terminal regulatory domain where protein-protein interaction occurs. SnRK1α2 is 78.9% similar to SnRK1α1 at the amino acid level. Solid lines, kinase domain; dotted lines, regulatory region. **d** Schematic of SnRK1α subunits. P = phosphorylation site (T175/6) in the catalytic domain; UBA = ubiquitin associated domain; KA1 = kinase-associated 1 motif. Numbers indicate amino acid positions in SnRK1α1.

a

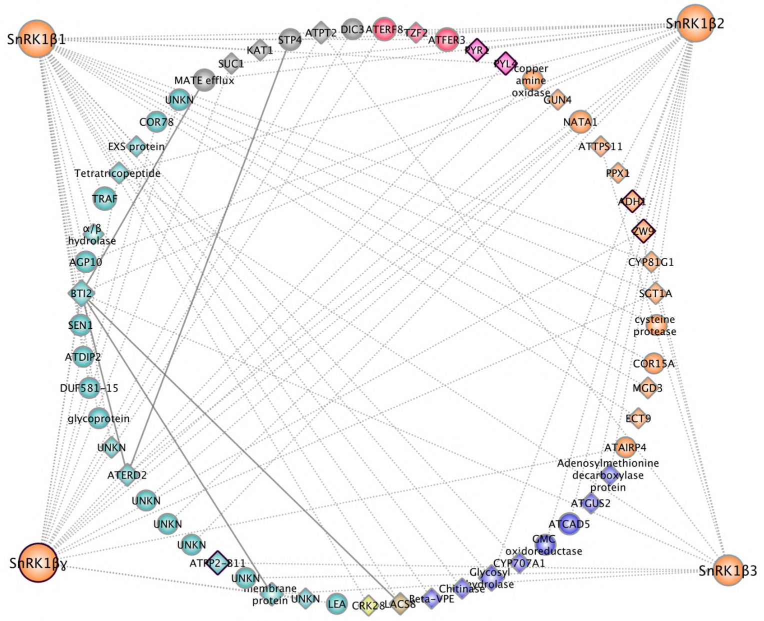

b

GO Molecular Function	Node	β/βɣ specific (57)
Unknown		39%
Metabolism		14%
Transcription		5%
Kinase		1.5%
Ligase		1.5%
Transporter		11%
Other		25%
Receptor		3%

c

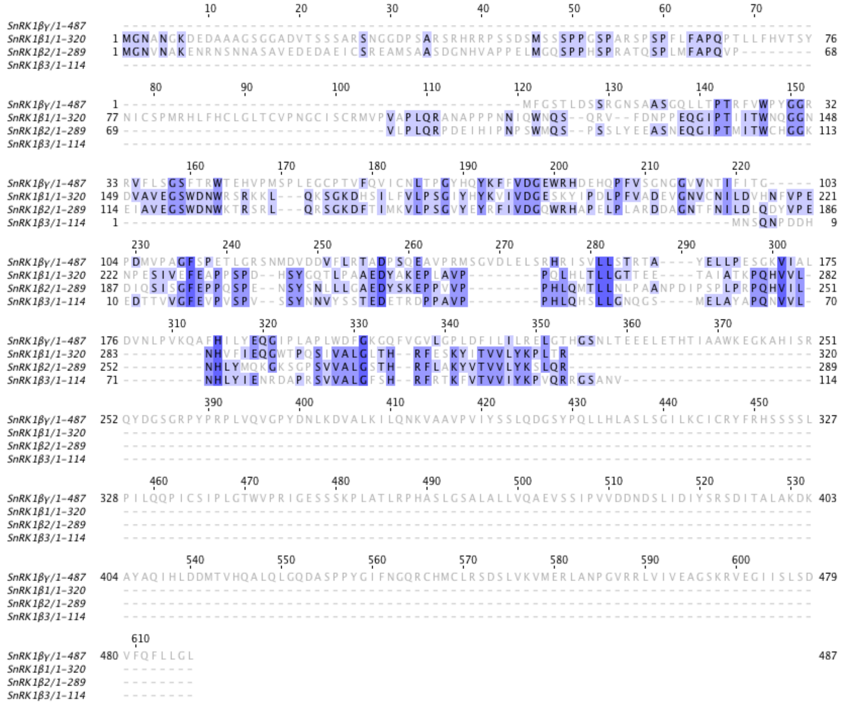

d

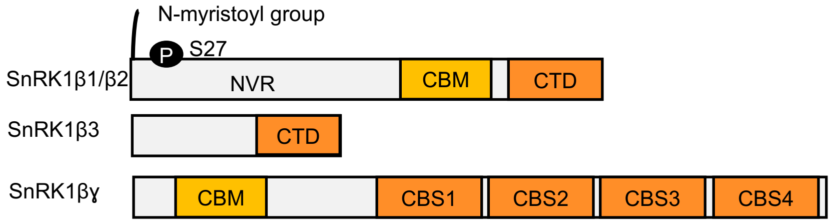

Supplementary Fig. 4. Overview of SnRK1β/βɣ interactors

a Overview of 57 SnRK1β/βɣ specific interactors (not part of the 48 core set shown in Fig 1b). Nodes are coloured based on GO molecular function. **b** Percentage distribution by GO molecular function for each set of β/βɣ-interactors. **c** ClustalOmega alignment of the SnRK1β/βɣ. Blue highlight denotes amino acid similarity. **d** Schematic of SnRK1β and βɣ subunits. NVR = N-terminal variable region; CBM = carbohydrate-binding module; CTD = C-terminal domain; CBS = cystathionine-β-synthase motif; P = phosphorylation site.

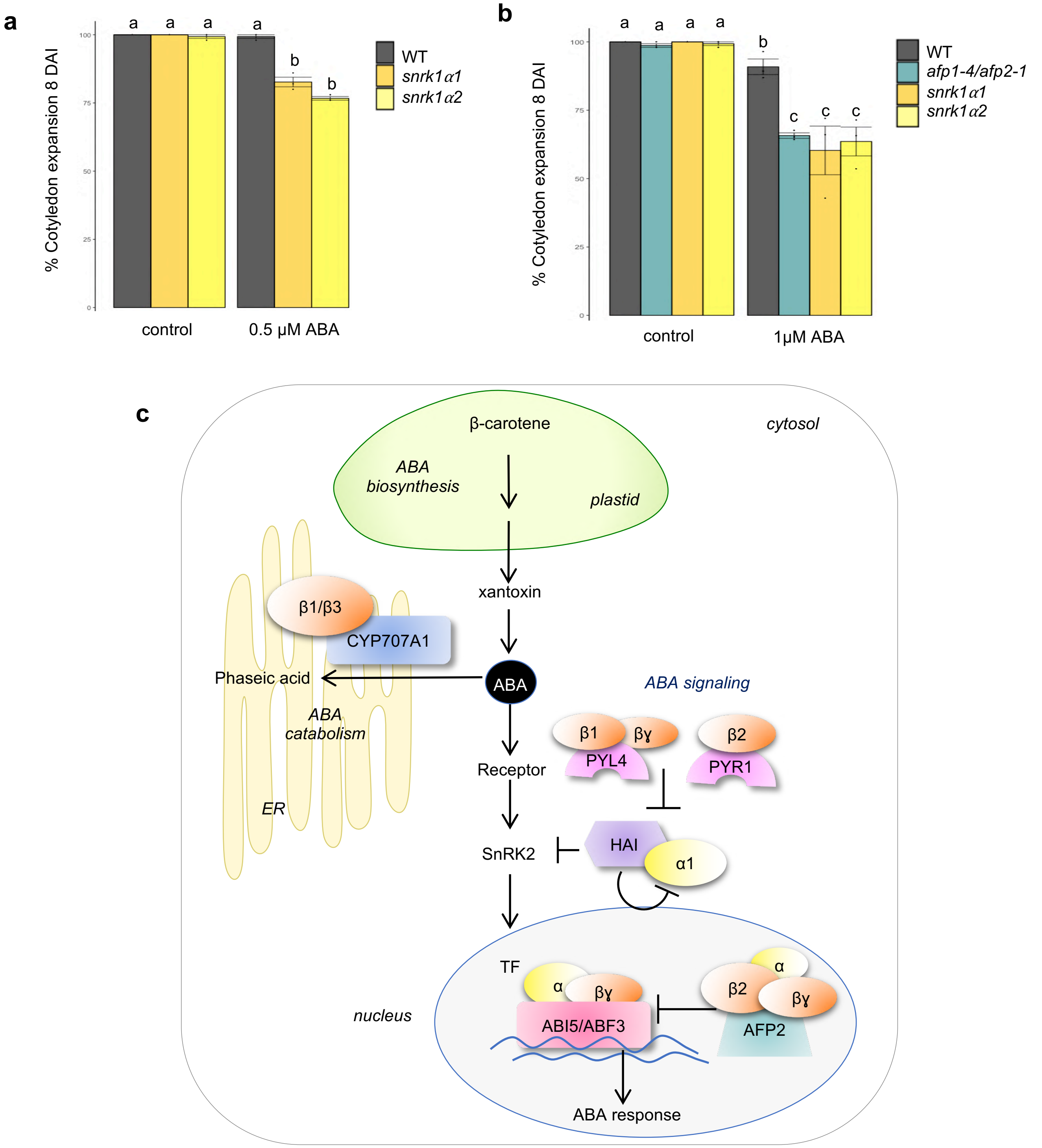

Supplementary Fig. 5. SnRK1 interactors impinge on the ABA synthesis and response pathways at multiple levels

a Quantification of cotyledon expansion of Wildtype (WT), *snrk1a1* and *snrk1a2* mutants on 0.5µM ABA at eight days after imbibition (DAI). **b** Quantification of cotyledon expansion of Wildtype (WT), *snrk1a1*, *snrk1a2* (Mair et al., 2015) and *afp1-4/afp2-1* (Garcia et al., 2008) mutants on 1µM ABA at eight days after imbibition (DAI). **b, c,** Mean of three independent biological samples ± SEM are shown; n=50 seedlings per biological sample. Dots represent the average of each biological replica. Error bars show standard error of the mean. Statistical significance calculated by ANOVA ($p < 0.01$) and posthoc Tukey test ($p < 0.05$). **c** Subunits of the SnRK1 complex interact with ABA catabolic enzymes (CYP707A1) and core ABA signaling components, including receptors (PYL4 and PYR1), PP2C phosphatases (HAI1 and AHG3), transcription factors (ABI5 and ABF3) as well as negative regulators (AFP2). Proteins are coloured based on their GO molecular function (see **Fig. 1**).

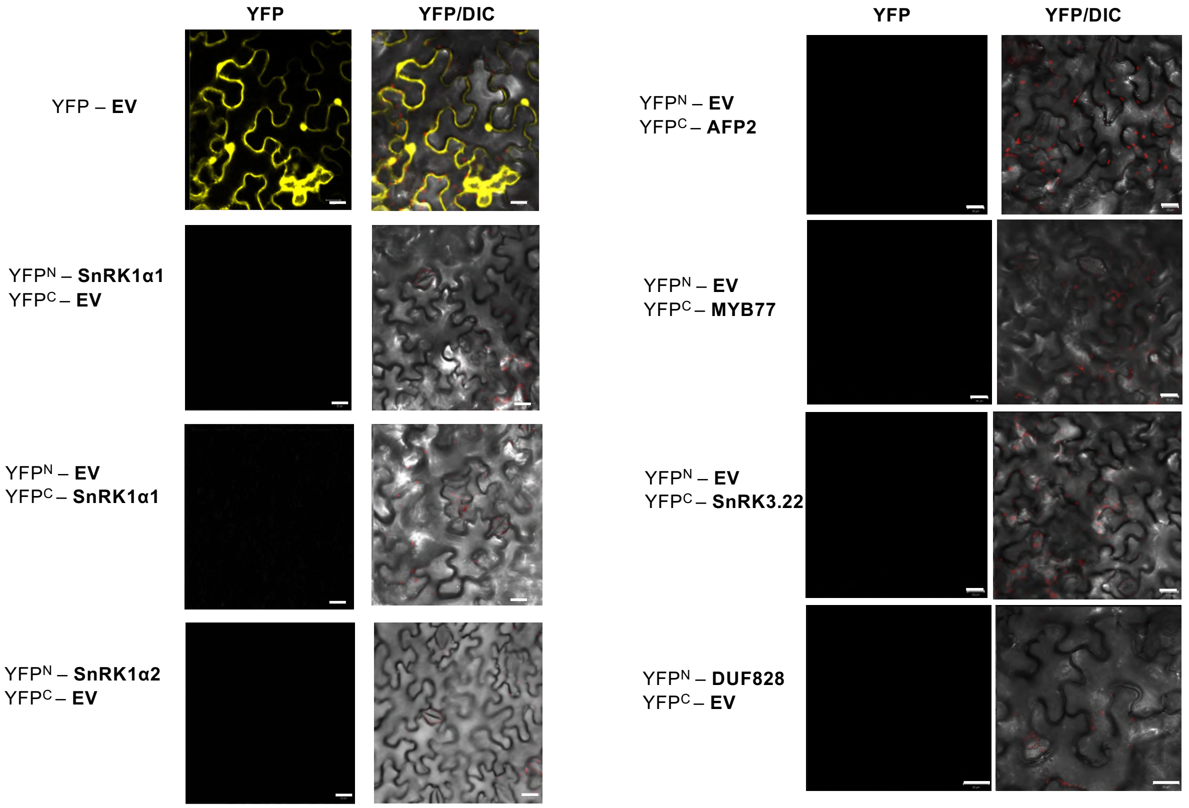

Supplementary Fig. 6. Negative controls for Bimolecular Fluorescence Complementation (BiFC)

Lack of interaction of SnRK1 catalytic subunits and SnIPs with the empty vector controls containing the N and C-terminus of YFP (YFPN-EV and YFPC-EV) was tested by BiFC. Localization of the empty vector containing the full-length YFP (YFP-EV) was used as positive control.

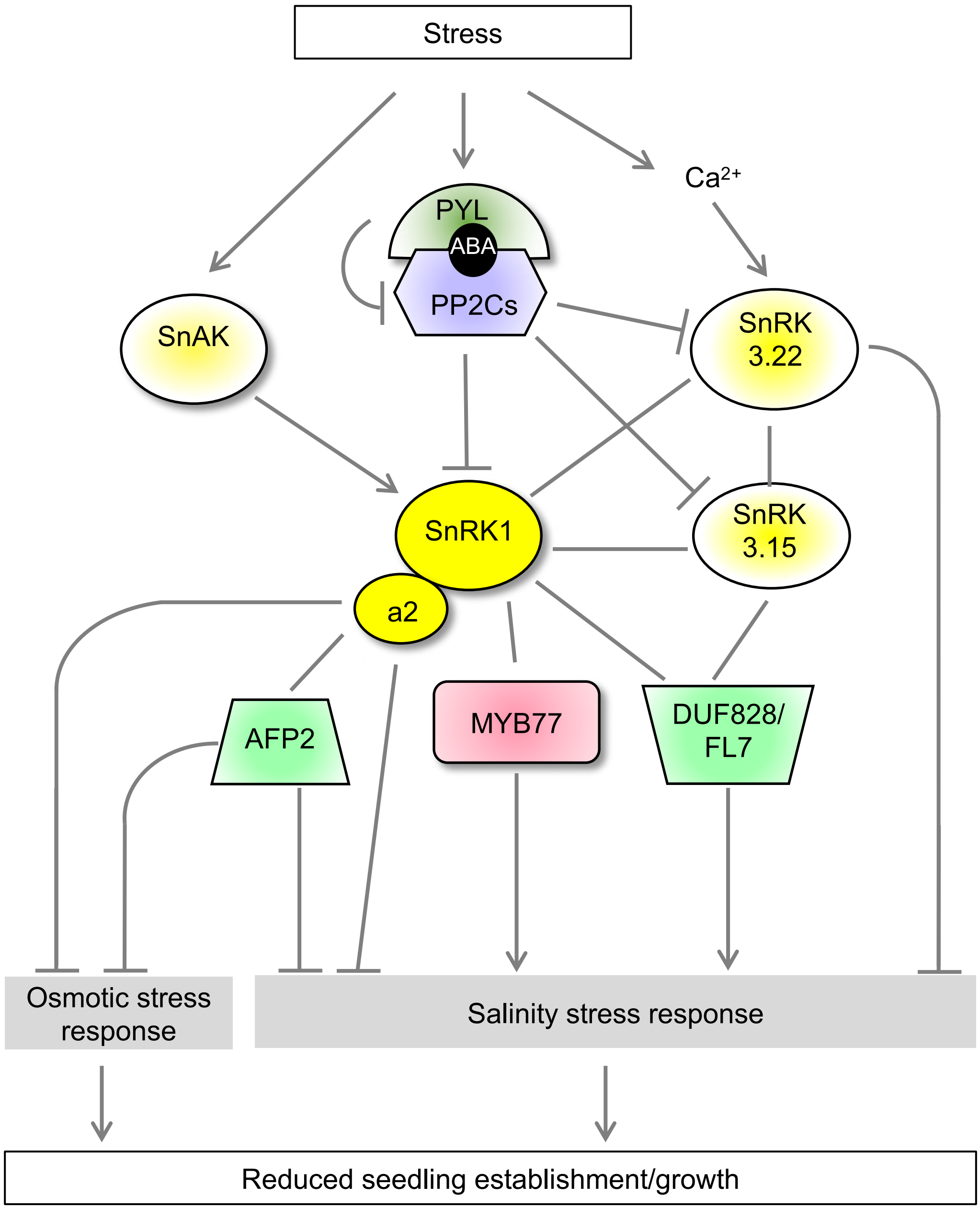

Supplementary Fig. 7. Model of the ABA responsive SnRK1 interaction network during abiotic stress response in *A. thaliana* during germination

ABA accumulates in response to stress, inhibiting clade-A PP2C phosphatases through PYL/RCAR receptors thus activating SnRK1 and SnRK3.15/3.22 kinases. Stress also activates SnRK1 through SnAKs and SnRK3 through calcium signaling. SnRK1 and SnRK3s interact with one another and may regulate each other function. SnRKs and PP2Cs interact with ABA and stress responsive SnIPs to modulate plant responses to abiotic stresses (salt, osmotic). Proteins are coloured based on GO molecular function (see **Fig. 1**). Arrows indicate positive regulation and T-lines indicate negative regulation.

Supplementary Table 1. *LacZ* and *LEU* reporter sensitivity determined with a positive reference set (PRS). Literature curated SnIPs-SnRK1 interactions were retested using the *LacZ* and *LEU* reporters (two replicates per reporter).

-	Lack of interaction
+	Positive interaction in one replicate
AA	Autoactivators of the respective reporter (as determined in Lumba et al., 2014).

Protein1	Protein2	PPI Method	Kinase substrate	Reference	*LacZ* (Primary screen)	*LacZ* (Retest)	*LEU* (Primary screen)	*LEU* (Retest)	PRS confirmed (either reporter)	PRS confirmed (both reporters)
ABI5	SnRK1α1	PD	yes	Bitrian, 2011	++	++	--	++	yes	yes
ABI5	SnRK1α2	PD	yes	Bitrian, 2012	++	++	--	++	yes	yes
ABF3	SnRK1α1	PD	yes	Bitrian, 2013	AA	AA	+-	++	yes	no
ABF3	SnRK1α2	PD	yes	Bitrian, 2014	AA	AA	++	++	yes	no
SnRK3.15 / CIPK14	SnRK1α1	Y2H (LacZ/His)	NA	Yan, 2014	--	++	+-	++	yes	yes
PP2CA / AHG3	SnRK1α1	Y2H; PD; IP	NA	Rodrigues, 2013	AA	AA	++	++	yes	no
DUF581-12/FLZ2	SnRK1α1	Y2H (His)	NA	Nietzsche, 2014; 2016	++	--	--	--	no	no
DUF581-12/FLZ2	SnRK1α2	Y2H (His)	NA	Nietzsche, 2016	--	--	--	--	no	no
ANAC002	SnRK1α1	Y2H (LacZ); PD	no	Kleinow, 2009	++	--	--	--	no	no
% positive interactions per reporter					4/6 = 67%	3/6 = 50%	2/9 = 22%	6/9 = 67%	6/9 = 67%	3/6 = 50%

Supplementary Table 2. *LacZ* and *LEU* reporter background determined with a random reference set (RRS).
Interactions retested with *LacZ*/*LEU* are shown in duplicates.

- (black) Positive interactions in the primary screen that were not retested
- (grey) Protein pairs that interacted in either or both reporters

Accession (Gene ID)	Gene name (gene symbol)	SnRK1α1 (At3g01090)	SnRK1α2 (At3g29160)	SnRK1β1 (At5g21170)	SnRK1β2 (At4g16360)	SnRK1β3 (At2g28060)	SnRK1βγ (At1g09020)
AT1G20450	ERD10	--/--	--/--	--/--	--/--	--/--	--/--
AT1G29330	ATERD2	--/+-	--/++	--/--		--/--	--/--
AT1G53170	ATERF8	--/--	--/--	--/--	--/--	--/--	
AT1G54130	RSH3	++/--		--/--	++/--	--/--	++/--
AT1G65390	ATPP2-A5	--/--	--/--	--/--	--/--	--/--	--/--
AT1G73920	Lipase		--/--	--/--	--/--	--/--	--/--
AT1G77120	ADH1	--/--	--/--	--/--			--/--
AT1G78680	ATGGH2	--/--	--/--	--/--	--/--	--/--	--/--
AT2G04350	LACS8	--/--	--/--		--/--	--/--	--/--
AT2G11810	MGD3	--/--	--/++		--/--	--/--	--/--
AT2G17740	DC1-domain		--/--	--/--	--/--	--/--	--/--
AT2G18700	ATTPS11	--/--	--/--	--/--	--/--		--/--
AT2G26170	CYP711A1	--/--	--/--	--/--	--/--	--/--	--/--
AT2G27860	AXS1	--	--/--	--/--	--/--	--/--	--/--
AT2G39030	NATA1	++/++	--/--	--/--	--/--	--/--	
AT2G46270	GBF3	--/--	--/--	--/--	--/--	--/--	--/--
AT3G13720	PRA8	--/--	--/--	--/--	--/--	--/--	--/--
AT3G56090	ATFER3	--/--	--/--		--/--	--/--	--/++
AT3G62150	ABCB21	--/--	--/--	--/--	--/--	--/--	--/--
AT4G00500	α/β hydrolase protein	--/--	--/--		--/--	--/--	--/+-
AT4G23700	CHX17	--/--	--/--	--/--	--/--	--/--	--/--
AT4G24960	HVA22D	++/+-	++/--	--/+-	++/+-	--/--	--/--
AT4G30960	SnRK3.14	--/--	--/+-	--/+-	--/--	--/--	--/+-
AT5G06870	PGIP2	--/--	--/--	--/--	--/--	--/--	--/--
AT5G07830	ATGUS2	--/--	--/--	--/--		--/--	--/--
AT5G46295	UNKN	--/--	--/--	--/--	--/--	--/--	--/--
AT5G49280	hydroxyproline-rich glycoprotein	--/--	--/--		--/--	--/--	--/--
AT5G67030	ABA1	--/--	--/--	--/--	--/--	--/--	--/+-

Supplementary Table 3. Literature curated interactions that tested positive in Lumba et al. (2014) Y2H screen. Interactions between protein pairs were scored with the *LacZ* and *LEU* reporters. Interactions that occurred only in the presence of ABA (PP2C - PYL/RCAR) are not listed. Yes, validation; no, lack of validation. PCA, protein complementation assay.

Protein1	Protein2	PPI method	Reference	Positive interactions
ABF3	AFP2	Y2H	Garcia et al., 2008	yes
ABF3	AFP4	Y2H	Garcia et al., 2008	yes
ABF3	ABF3	Y2H; BiFC	Lynch et al., 2012	no
ABF3	ABI5	Y2H; BiFC	Lynch et al., 2012	yes
ABI5	AFP2	Y2H	Garcia et al., 2008	yes
ABI5	AFP4	Y2H	Garcia et al., 2008	yes
AFP2	AFP4	Y2H	Garcia et al., 2008	no
AFP2	AFP2	Y2H	Garcia et al., 2008	no
AFP4	AFP4	Y2H	Garcia et al., 2008	no
AHG3	ABF3	Y2H	Lynch et al., 2012	no
PYL8	MYB77	Y2H; PCA	Zhao et al., 2014	yes
SnRK3.22	ABI5	Y2H; PCA	Zhou et al., 2015	no
ABI1	ABF3	Y2H	Lynch et al., 2012	no
ABI1	SnRK3.15/PKS24/CIPK14	Y2H	Ohta et al., 2003	yes
ABI1	ATHB6	Y2H; in vitro binding	Himmelbach et al., 2002	no
			% positive interactions	7/15 = 47%

Garcia, M. E., Lynch, T., Peeters, J., Snowden, C., & Finkelstein, R. (2008). A small plant-specific protein family of ABI five binding proteins (AFPs) regulates stress response in germinating Arabidopsis seeds and seedlings. Plant Molecular Biology 67, 643-658.

Lynch T, Erickson BJ, Finkelstein RR. Direct interactions of ABA-insensitive(ABI)-clade protein phosphatase(PP)2Cs with calcium-dependent protein kinases and ABA response element-binding bZIPs may contribute to turning off ABA response. Plant Mol Biol. 2012 Dec;80(6):647-58.

Zhao Y, Xing L, Wang X, Hou YJ, Gao J, Wang P, Duan CG, Zhu X, Zhu JK. The ABA receptor PYL8 promotes lateral root growth by enhancing MYB77-dependent transcription of auxin-responsive genes. Sci Signal. 2014 Jun 3;7(328):ra53.

Zhou X, Hao H, Zhang Y, Bai Y, Zhu W, Qin Y, Yuan F, Zhao F, Wang M, Hu J, Xu H, Guo A, Zhao H, Zhao Y, Cao C, Yang Y, Schumaker KS, Guo Y, Xie CG. SOS2-LIKE PROTEIN KINASE5, an SNF1-RELATED PROTEIN KINASE3-Type Protein, is Important for Abscisic Acid Responses in Arabidopsis through Phosphorylation of ABSCISIC ACID-INSENSITIVE5. Plant Physiol. 2015 Jun;168(2):659-76.

Himmelbach A, Hoffmann T, Leube M, Höhener B, Grill E. Homeodomain protein ATHB6 is a target of the protein phosphatase ABI1 and regulates hormone responses in Arabidopsis. EMBO J. 2002 Jun 17;21(12):3029-38.

Ohta M, Guo Y, Halfter U, Zhu JK. A novel domain in the protein kinase SOS2 mediates interaction with the protein phosphatase 2C ABI2. Proc Natl Acad Sci U SA. 2003 Sep 30;100(20):11771-6.

Supplementary Table 4. Retest of high confidence interactions (HCI) by Y2H. 132 HCI were rested. Interactions retested with *LacZ* / *LEU* are shown in duplicates. Positive interaction scored as 2/2 plates for each reporter.

- *LacZ*
- *LEU*
- *LacZ* & *LEU*
- Negative interactions in the primary screen, which were not retested
- Interactions that were not validated in either reporter (false positive)

Accession (Gene ID)	Gene name (gene symbol)	SnRK1α1 (At3g01090)	SnRK1α2 (At3g29160)	SnRK1β1 (At5g21170)	SnRK1β2 (At4g16360)	SnRK1β3 (At2g28060)	SnRK1βγ (At1g09020)
AT1G07870	protein kinase	--/--	--/--				--/--
AT1G10585	bHLH/AIB1	++/+-	++/--		++/++		
AT1G16640	AP2/B3-like TF	++/++	++/++	++/++	++/+-	++/++	++/++
AT1G60600	ABC4		++/++	++/++	++/++	++/++	++/++
AT1G74930	ORA47	++/++	++/--			--/++	--/++
AT1G76210	DUF241	++/++					++/--
AT1G77450	ANAC032	++/--	++/++	++/--	++/--	++/--	++/--
AT1G78070	Transducin/WD40 repeat-like		++/--	++/++	++/++	++/++	--/++
AT2G22430	ATHB6	++/++	++/++	++/++	++/--	++/++	++/++
AT2G30360	SnRK3.22	++/++	++/++		++/+-		++/--
AT2G39050	ARATHEULS3	++/++			++/++	--/++	--/++
AT2G46450	CNGC12	++/--	++/++				++/++
AT2G46640	TAC1	--/--				--/--	
AT2G47460	MYB12	--/+-		--/--			
AT3G15790	MBD11		--/--		--/--		
AT3G16150	ASPGB1	++/++	++/++	++/++	++/++	++/++	++/++
AT3G44450	UNKN	++/++	++/++	++/++	++/++	++/++	++/++
AT3G48510	DIG1	++/--	++/--	--/--	++/--		--/--
AT3G50060	MYB77	++/++			--/--		++/--
AT3G59050	ATPAO3	--/--					++/++
AT4G16670	DUF828/FL7	++/++	++/++	--/++		--/++	++/++
AT4G23050	MAP3Kδ4	++/++	++/+-		++/+-		--/--
AT4G25160	PUB35		++/+-	+-/+-	++/++	--/--	++/++
AT4G36410	UBC17	--/++	-+/--				++/++
AT5G01600	ATFER1		++/--				--/++
AT5G14760	AO	++/++	++/+-	--/--	++/--		--/+-
AT5G24230	α/β hydrolase protein	++/--		--/--			
AT5G40590	DC1 domain	++/++	+-/++	++/+-	++/+-	++/++	--/+-
AT5G46410	SSP4	--/--				--/+-	
AT5G47220	ATERF2	--/++	--/++	--/++			
AT5G51190	ERF105		--/--	--/--	--/--		
AT5G53590	SAUR30	--/--					++/--
AT5G54230	MYB49	++/+-	++/--	--/--	--/+-	--/--	--/--
AT5G54470	BBX29	++/++	--/+-				--/++
AT5G55560	protein kinase		++/++		++/+-		

Supplementary Table 5. List of interactors shared between SnRK1 and SnRK3. Black, no interaction; (+) interaction.

Accession (Gene ID)	Gene name (gene symbol)	SnRK1α1	SnRK1α2	SnRK3.15	SnRK3.22	SnRK3.14
AT1G10585	AIB1	+	+	+	+	
AT2G22430	HB6	+	+	+	+	
AT5G43520	DC1-domain		+	+	+	
AT5G54230	MYB49	+	+	+	+	
AT1G74930	ORA47	+	+		+	
AT2G36270	ABI5	+	+		+	
AT3G48510	DIG1	+	+		+	
AT5G48240	UNKN		+		+	
AT1G77450	ANAC032	+	+	+		
AT3G22420	WNK2		+	+		
AT4G16670	DUF828/FL7	+	+	+		
AT1G52880	ANAC018	+		+	+	
AT1G52890	ANAC019	+		+	+	
AT2G47460	MYB12	+		+	+	
AT5G37260	CIR1	+		+	+	
AT5G46410	SSP4	+		+	+	
AT3G51660	MIF	+			+	
AT1G73920	Lipase	+		+		
AT2G15970	COR413-PM1	+		+		
AT2G39050	EULS3	+		+		

Supplementary Table 6. Primer list used for cloning SnRK1 constructs by Gateway.

**SnRK1a1_F(BP)**	GGGGACAAGTTTGTACAAAAAAGCAGGCTAGTTCAAACGAGTAGATG	Primers used for PCR amplification of SnRK1 subunits to use in BP reaction
**SnRK1a2_R(BP)**	GGGGACCACTTTGTACAAGAAAGCTGGGTTGAGGACTCGGAGCTGAGC	
**SnRK1a2_F(BP)**	GGGGACAAGTTTGTACAAAAAAGCAGGCTTCATGGATCATTCATCAAATAGATTTGGCA	
**SnRK1a2_R(BP)**	GGGGACCACTTTGTACAAGAAAGCTGGGTCTCAGATCACACGAAGCTCTGTA	
**SnRK1by_F(BP)**	GGGGACAAGTTTGTACAAAAAAGCAGGCTTCATGTTTGGTTCTACATTGGATAGCA	
**SnRK1by_R(BP)**	GGGGACCACTTTGTACAAGAAAGCTGGGTCTCAAAGACCGAGCAGGAATTGGAA	
**SnRK1b1_F(BP)**	GGGGACAAGTTTGTACAAAAAAGCAGGCTTCATGGGAAATGCGAACGGCAAAGA	
**SnRK1b1_R(BP)**	GGGGACCACTTTGTACAAGAAAGCTGGGTCTTACCGTGTGAGCGGTTTGTAGAG	
**SnRK1b2_F(BP)**	GGGGACAAGTTTGTACAAAAAAGCAGGCTTCATGGCTATGTCTGCTGCTTCTGAT	
**SnRK1b2_R(BP)**	GGGGACCACTTTGTACAAGAAAGCTGGGTCTCACCTCTGCAGGGATTTGTAGAG	
**SnRK1b3_F(BP)**	GGGGACAAGTTTGTACAAAAAAGCAGGCTTCATGAACAGTCAAAATCCTGATGAT	
**SnRK1b3_R(BP)**	GGGGACCACTTTGTACAAGAAAGCTGGGTCTCAAACATTGGCGCTCCCTCTTCT	
